# Memantine prevents memory consolidation failure induced by soluble beta amyloid in rats

**DOI:** 10.3389/fnbeh.2014.00332

**Published:** 2014-09-19

**Authors:** Paolo Tucci, Emanuela Mhillaj, Maria Grazia Morgese, Marilena Colaianna, Margherita Zotti, Stefania Schiavone, Maria Cicerale, Viviana Trezza, Patrizia Campolongo, Vincenzo Cuomo, Luigia Trabace

**Affiliations:** ^1^Department of Experimental and Clinical Medicine, Faculty of Medicine, University of FoggiaFoggia, Italy; ^2^Department of Pathology and Immunology, University of GenevaGeneva, Switzerland; ^3^Department of Mental Health and Psychiatry, Geneva University Hospital and University of GenevaGeneva, Switzerland; ^4^Department of Sciences, University “Roma Tre”Rome, Italy; ^5^Department of Physiology and Pharmacology, La Sapienza, University of RomeRome, Italy

**Keywords:** soluble beta amyloid, glutamate, short term memory, long term memory, memantine

## Abstract

It has been well documented that β-amyloid (Aβ) peptide accumulation and aggregation in the brain plays a crucial role in the pathophysiology of Alzheimer’s disease (AD). However, a new orientation of the amyloid cascade hypothesis has evidenced that soluble forms of the peptide (sAβ) are involved in Aβ-induced cognitive impairment and cause rapid disruption of the synaptic mechanisms underlying memory. The primary aim of this study was to elucidate the effects of sAβ, acutely injected intracerebrally (i.c.v., 4 μM), on the short term and long term memory of young adult male rats, by using the novel object recognition task. Glutamatergic receptors have been proposed as mediating the effect of Aβ on synaptic plasticity and memory. Thus, we also investigated the effects of sAβ on prefrontal cortex (PFC) glutamate release and the specific contribution of N-methyl-D-aspartate (NMDA) receptor modulation to the effects of sAβ administration on the cognitive parameters evaluated. We found that a single i.c.v. injection of sAβ 2 h before testing did not alter the ability of rats to differentiate between a familiar and a novel object, in a short term memory test, while it was able to negatively affect consolidation/retrieval of long term memory. Moreover, a significant increase of glutamate levels was found in PFC of rats treated with the peptide 2 h earlier. Interestingly, memory deficit induced by sAβ was reversed by a NMDA-receptor antagonist, memantine (5 mg/kg i.p), administered immediately after the familiarization trial (T1). On the contrary, memantine administered 30 min before T1 trial, was not able to rescue long term memory impairment. Taken together, our results suggest that an acute i.c.v. injection of sAβ peptide interferes with the consolidation/retrieval of long term memory. Moreover, such sAβ-induced effect indicates the involvement of glutamatergic system, proposing that NMDA receptor inhibition might prevent or lead to the recovery of early cognitive impairment.

## Introduction

Extensive research on the neurotoxic role of beta amyloid (Aβ) in its fibrillar form has demonstrated that it accumulates in the brain as extracellular insoluble plaques around neurons and glia (Kowalewski and Holtzman, 1999; Mucke and Selkoe, 2012). Later, this view changed when it was recognized that in Alzheimer’s disease (AD) patients, cognitive impairment is poorly correlated with counts of “senile plaques” in cerebral gray matter either in patients (Blennow et al., 1996; Berg et al., 1998; Giannakopoulos et al., 2003, 2009) or in animal models (Puoliväli et al., 2002; Christensen et al., 2008; Watanabe et al., 2009; Zhang et al., 2012). More recently, in support of this, the initial etiopathogenetic hypothesis has been questioned by the failure of several clinical trials testing drugs targeting Aβ accumulation in the brain (Mangialasche et al., 2010).

Instead, a new orientation of the amyloid cascade hypothesis appeared and more recent studies suggested that a stronger interrelationship exists between the presence of soluble beta amyloid (sAβ) and dementia severity. Soluble Aβ oligomers were recently reported to be dramatically increased in the soluble fraction of AD brain extracts (Sokolow et al., 2012), and they have been found to correlate with disease progression or decline in synaptic density in AD patients (Lue et al., 1999; McLean et al., 1999). In this scenario, soluble oligomeric, globular and protofibrillar amyloid species, rather than fibrils, could be considered the first toxic species and are hypothesized to contribute to synaptic failure and early cognitive loss in AD (Arendt, 2009). In this regard, the role of sAβ in the pathophysiology of AD still remains a matter of intense research at present. Interestingly, impaired neuronal functions have been directly attributed to soluble oligomeric forms of Aβ, even before the appearance of overt signs of neurotoxicity (Lesné et al., 2006; Haass and Selkoe, 2007). Moreover, sAβ has been found to cause rapid disruption of the synaptic mechanisms underlying memory. It has been demonstrated that sAβ oligomers, directly extracted from the cerebral cortex of subjects with AD, were able to disrupt the memory of a learned behavior in normal rats (Shankar et al., 2008). However, very few studies have focused on the investigation of the effects of defined sequences of sAβ on memory. In this regard, transgenic mice models of AD, although covering several aspects of the disease, do not permit an accurate identification of Aβ species responsible of memory loss, being the peptide expressed either in soluble or insoluble forms in the brain. This characteristic does not allow to determine which specific Aβ species is responsible for the detrimental effects. On the other hand, peptide injection in the rat’s brain might be considered as a valid alternative to transgenic animals to evaluate the effects of an increase of sAβ species in the brain without presence of plaques. Then, to date, the comprehension of the specific role of sAβ on different temporal forms of memory remains an urgent need. To further investigate the specific effects of sAβ, we recently developed a simple and reliable rat model of memory impairment induced by an acute i.c.v. injection of sAβ1-42 (Trabace et al., 2007; Colaianna et al., 2010). We demonstrated that sAβ significantly affected behavior that rely on stressful conditions. In particular, sAβ-treated rats were not able to retrieve the learned behavior, fear conditioned, in a passive avoidance task (Morgese et al., 2014).

In order to evaluate if sAβ-treated rats may differently respond to behavioral cognitive tasks that do not involve stressful situations, we used an animal paradigm that is not demanding for the animals, but is based on the natural propensity of rats to explore novel situations. A simple and reliable task widely used for the investigation of memory is the one-trial novel object recognition (NOR) test. This procedure typically consists of three phases: habituation, familiarization, and finally the test phase. Rats naturally tend to approach and to explore novel over familiar objects. Such task relies on spontaneous animal behavior without the need neither for positive reinforcers nor for stressor elements, such as water or food deprivation, electric foot-shock or aversive environments. In particular, the ability of recognizing a previously presented stimulus varies according to the delay between the familiarization and the test phase. Based on this, the NOR test is very useful to study short-term and long-term memory (Ennaceur and Delacour, 1988; Taglialatela et al., 2009). This is possible by manipulating the amount of time rats must retain memory of the sample objects presented during the familiarization phase. In particular, short term memory is represented by the process that maintains a representation of information for a short period of time, and it is available for posterior use, while the long-term memory requires stabilization to persist. This process implies a reorganization of the already established memories, allowing then incorporation of new information (Clarke et al., 2010). In this regard, early studies carried out in senescence-accelerated-prone 8 (SAMP8) mouse strain, presenting cognitive deficiencies that could be related to the accumulation of amyloid aggregates, have shown a functional alteration in the hippocampal-prefrontal circuits which is related to the object recognition deficit (Lopez-Ramos et al., 2012).

The current study was designed to address on which phase of the memory process sAβ exerts its effects. We evaluated the effects of sAβ, acutely injected intracerebrally, on the short term and long term memory of young adult male rats. Since the training-testing interval is extremely informative regarding the mechanism of action underlying the type of memory impairment, we chose two different experimental paradigms, especially well-suited to study short term memory (1 min retention interval, NOR1) and long term memory (24 h retention interval, NOR2).

Literature data point out that soluble forms of the peptide, in particular oligomers ranging from dimers and trimers to dodecamers, are involved in Aβ-induced cognitive impairment and are capable of blocking long-term potentiation (LTP) and the reversal of long term depression (LTD; Lambert et al., 1998; Wilcox et al., 2011). Moreover, in Aβ-based model of AD, it has been found a predominant susceptibility of glutamatergic synapses (Canas et al., 2014). In addition, a direct application of sAβ oligomers or fragments to cultured hippocampal neurons was found to rapidly trigger sustained calcium entry through N-methyl-D-aspartate (NMDA) receptors that was prevented by antagonists of NMDA receptors (Kelly and Ferreira, 2006; De Felice et al., 2007) and to enhance NMDA-evoked cell firing rate (Molnár et al., 2004). Moreover, fragments of Aβ have been shown to bind glycine and glutamate recognition sites on NMDA receptors (Cowburn et al., 1997). Therefore, a further aim of the present study was to examine the effects of sAβ on glutamate release and the specific contribution of NMDA receptor modulation to the cognitive deficits associated with sAβ administration. To this end, we tested the effects of NMDA receptor inhibition on memory disruption in rats i.c.v. injected with sAβ.

## Materials and methods

### Animals

All experiments were conducted in young male Wistar rats (Harlan S. Pietro al Natisone, Udine, Italy) weighing 250–300 g. They were group-housed at constant room temperature (22 ± 1°C) and relative humidity (55 ± 5%) under a 12-h light/dark cycle (lights on from 7:00 AM to 7:00 PM) for at least 7 days before the experiments. Food and water were available *ad libitum*. Procedures involving animals and their care were conducted in conformity with the institutional guidelines in compliance with national (D.L. 116/92) and international laws and policies (EEC Council Directive 2010/63/EU; Guide for the Care and Use of Laboratory Animals, U.S. National Research Council, 1996). All efforts were made to minimize the number of animals used and their suffering.

### Surgery

Surgery procedures were performed as previously described (Colaianna et al., 2010). Briefly, rats were anesthetized with 3.6 ml/kg Equithesin intraperitoneally (i.p.; composition: 1.2 g sodium pentobarbital; 5.3 g chloral hydrate; 2.7 g MgSO_4_; 49.5 ml propylene glycol; 12.5 ml ethanol and 58 ml distilled water) and secured in a stereotaxic frame (David Kopf Instruments, Tujunga, CA, USA). The skin was shaved, disinfected and cut with a sterile scalpel to expose the skull in order to perform the procedures described below.

### Soluble Aβ1-42 administration

The sAβ peptide was obtained from Tocris (Bristol, UK). All solutions were freshly prepared. Peptide was dissolved in sterile double-distilled water (vehicle) at a concentration of 4 μM as previously described (Colaianna et al., 2010). On the day of surgery, bilateral 23 gauge guide cannulae were implanted using the following coordinates relative to Bregma: AP = −0.5 mm, ML = +1.2 mm, DV = −3.0 mm with the incisor bar set at −3.3 mm, according to a stereotaxic atlas (Paxinos and Watson, 1998). The day after surgery procedure, intraventricular injections were made using injection needles (30-gauge stainless steel tubing; Cooper’s Needles, Birmingham, UK) which extended an additional 0.2 mm below the guide cannulae (total depth 3.2 mm). Soluble Aβ (5 μl) was delivered through a 25 μl Hamilton microsyringe at 2 μl/min infusion rate for duration of 2.5 min. Control rats were injected with vehicle only, because reverse sAβ42-1, used in preliminary experiments, had no effect on the measured parameters and was indistinguishable from vehicle alone (unpublished observations). The injection needle was left in place for additional 5 min to prevent reflux of the solution. The injection placement of needle track was verified at the time of dissection. All experimental procedures were performed 2 h after i.c.v. administration (sham-operated or sAβ-treated groups).

### Other chemicals

Memantine hydrochloride (3,5-dimethyl-1-adamantanamine hydrochloride, 3,5-dimethylamantadine hydrochloride) was purchased from Sigma-Aldrich Chemical Co. (Milan, Italy). It was dissolved in 1 ml/kg of 0.9% NaCl (saline) and administered intraperitoneally at a dose of 5 mg/kg.

### Novel Object Recognition (NOR) test

During the NOR test rats were submitted to two habituation sessions where they were allowed 5 min to explore the apparatus (circular arena, 75 cm diameter).

**Figure 1:**
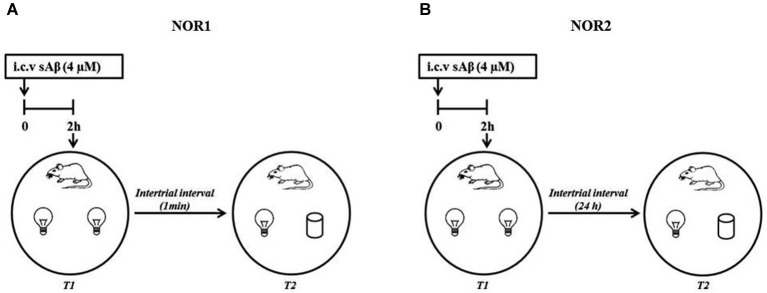
**Schematic representation of experimental procedure (A) NOR1 protocol according to Giustino et al. (1996); (B) NOR2 protocol according to Balducci et al. (2010)**.

The NOR1 test was performed according to Giustino et al. (1996). Briefly, 24 h after last habituation, a session of two 3-min trials separated by a 1-min intertrial interval (retention interval) was carried out (Figure 1A).

The NOR2 test was performed according to Balducci et al. (2010). Briefly, 24 h after last habituation, a session of two 10-min trials separated by a 24 h intertrial interval (retention interval) was carried out (Figure 1B). In both experimental conditions (NOR1 and NOR2), 2 h before the first trial (T1, training session), rats received i.c.v injection of sAβ solution or vehicle. Then, animals were exposed to two identical objects (white glasses or light bulbs). During the second trial (T2, testing session), rats were exposed to one familiar (F) object and the second was replaced by a new object (N) differently shaped. In a different session using the NOR2 protocol, memantine was administered i.p. (5 mg/kg) 30 min before or immediately after T1 trial.

In both protocols, during each trial, objects were placed in an equidistant position between the center and the wall of the arena. From rat to rat, the position of the two objects was counterbalanced and randomly permuted during T2. At the beginning of each trial, rats were placed near the center of the arena with their heads oriented in the opposite direction to the objects. Exploration of the objects was defined as sniffing or touching the object with the nose. Turning around or sitting on the object was not considered as exploration (Ennaceur and Delacour, 1988). Object exploration was quantified as: exploratory activity, total time spent exploring both objects during each trial (T1 and T2); index of discrimination expressed as ratio between the net time spent exploring the new (N−F) over the total exploration time (N−F/N+F). Objects and arena were carefully cleaned between each session to avoid olfactory confounding stimuli. Preliminary tests showed that none of the objects used in our experiments evoked innate preference.

### Microdialysis procedure

Animals were deeply anesthetized as reported above and placed on a stereotaxic apparatus (David Kopf Instruments, Tujunga, CA). One horizontal custom-constructed microdialysis probe (AN69 HospalS.p.A; 20 kDa cut-off, 6 mm length) was implanted aiming at the prefrontal cortex (PFC). The coordinates, measured from the interaural line, were AP = +10.7 mm and DV = +8.0 mm according to the atlas (Paxinos and Watson, 1998). The microdialysis probe was fixed to the skull with stainless steel screws and methylacrylic cement. Twenty four hours after surgery, the microdialysis probe was perfused with artificial CSF solution (NaCl 145 mmol/L, KCl 2.7 mmol/L, CaCl_2_ 2H_2_O 1.2 mmol/L, MgCl_2_ 6H_2_O 1 mmol/L, Na_2_HPO_4_ 2 mmol/L, pH 7.4) at a constant flow rate of 2 μl/min. Perfusates were collected every 20 min into mini-vials. After a wash-out period of 2 h, four samples were collected to determine the baseline levels of glutamate (no more than 10% difference among four consecutive samples). Glutamate concentrations were detected and quantified by high performance liquid chromatography (HPLC).

### Quantification of glutamate by HPLC

Glutamate concentrations were determined by HPLC using ODS-3 column (150 × 4.6 mm, 3 μm; INERTSIL) with fluorescence detection after derivatization with ophthalaldehyde/mercaptopropionic acid (emission length, 4.60 nm; excitation length, 3.40 nm). The mobile phase gradient consisted of 50 mM sodium acetate buffer, pH 6.95, with methanol increasing linearly from 2 to 30% (v/v) over 40 min. The flow rate was maintained by a pump (JASCO, Tokyo, Japan) at 0.5 ml/min. Results were analyzed by Borwin software (version 1.50; Jasco) and substrate concentration was expressed as μM.

### Statistical analysis

All statistical analyses were performed using Graph Pad® 5.0 for Windows. Data were tested for normality by the selection of parametric and non-parametric tests. Data were analyzed by a Two-way analysis of variance for repeated measures (Two-way RM ANOVA) followed by a Bonferroni’s multiple comparison test or Student’s *t*-test, as required. Differences were considered significant only when *P*-values were less than 0.05.

## Results

### Effects of acute i.c.v. injection of sAβ on short term memory (NOR1)

**Figure 2:**
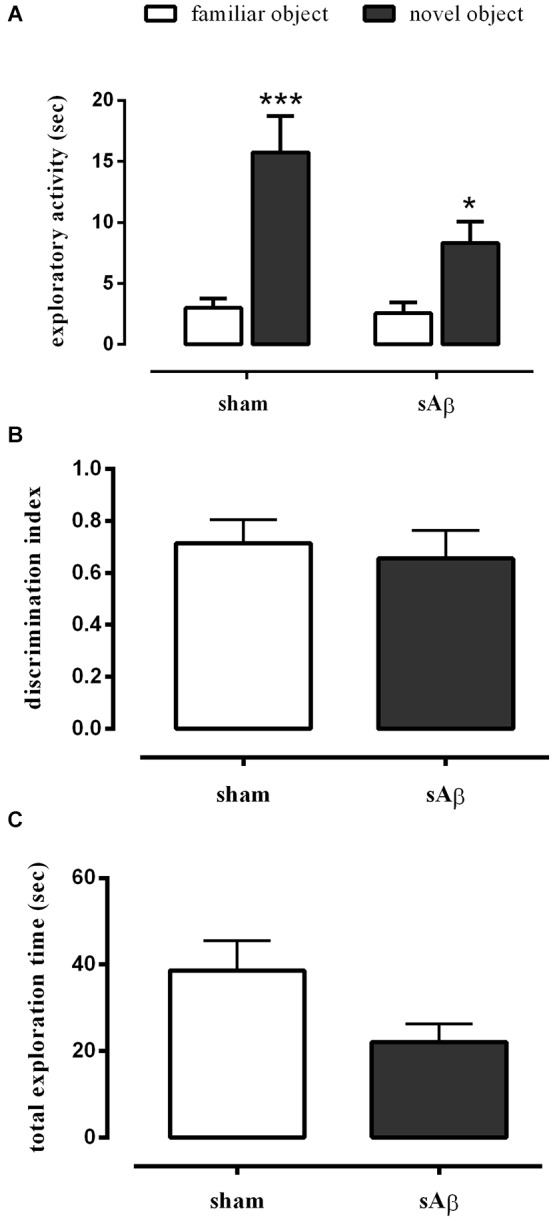
**(A)** Exploratory activity of novel and familiar objects during T2, **(B)** discrimination index and **(C)** total exploration time in NOR1 test. Animals received i.c.v. injection of vehicle (5 μl; sham) or sAβ (4 μM, 5 μl) 2 h before training (T1) and tested 1 min after (T2). Data are expressed as mean ± SEM of total time in s (*n* = 8 sham; *n* = 9 sAβ-treated group). (Two-way RM ANOVA followed by Bonferroni’s multiple comparisons test ***P < 0.0001 vs. familiar object in sham group and *P < 0.05 vs. familiar object in sAβ-treated group).

Our data showed that either sham- or sAβ-treated rats exhibited normal performance in the NOR1 test, with a significant difference in total time spent exploring the new object rather than the familiar one during the test (Two-way RM ANOVA followed by Bonferroni’s multiple comparisons test: *Ft*_(1,15)_ = 41.54, *P* < 0.0001; Figure 2A). Moreover, the preference between the different objects (discrimination index) during testing did not change significantly between experimental groups showing that sAβ, injected 2 h before training, did not affect short term memory for the familiar object (*t*-test: n.s.; Figure 2B). As shown in Figure 2C, no statistical differences were found in total exploration time, during both trials (T1 and T2), between sham and sAβ-treated rats (*t*-test: n.s.).

### Effects of acute i.c.v. injection of sAβ on long term memory (NOR2)

**Figure 3:**
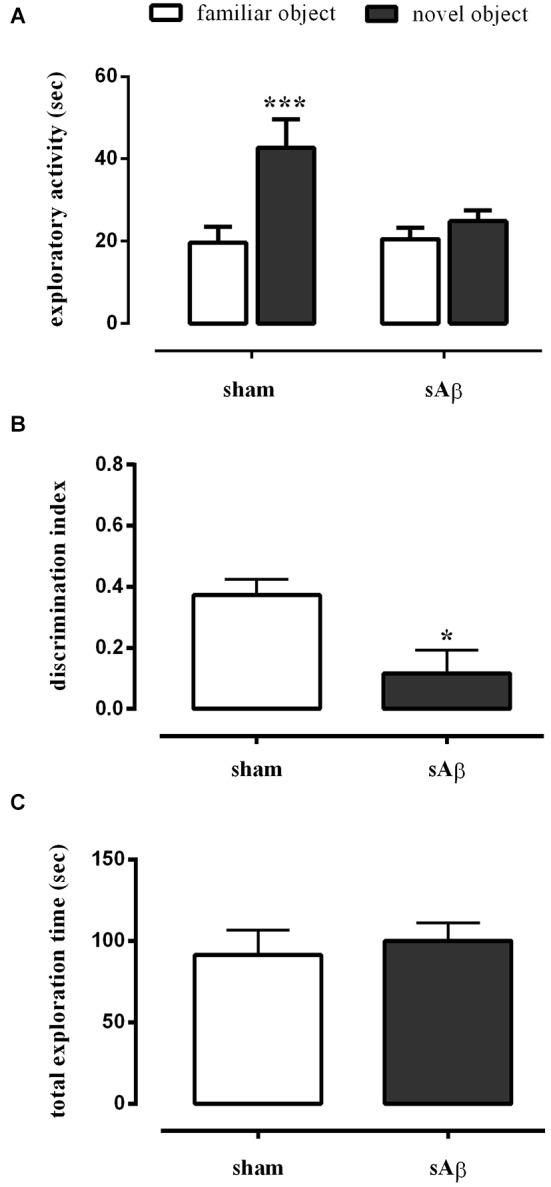
**(A)** Exploratory activity of novel and familiar objects during T2, **(B)** discrimination index and **(C)** total exploration time in NOR2 test. Animals received i.c.v. injection of vehicle (5 μl; sham) or sAβ (4 μM, 5 μl) 2 h before training (T1) and tested 24 h later (T2). Data are expressed as mean ± SEM of total time in s (*n* = 6 sham; *n* = 12 sAβ-treated group). (Two-way RM ANOVA followed by Bonferroni’s multiple comparisons test ***P < 0.001 vs. familiar object; *t*-test *P < 0.05 vs. sham).

As shown in Figure 3A, sAβ-treated rats failed to acquire the object recognition memory. Statistical analysis revealed a significant difference in exploratory activity between the experimental groups (Two-way RM ANOVA: *Ft*_(1,16)_ = 28.52, *P* < 0.0001). In particular, sAβ-treated rats were not able to recognize the novel object, while sham-operated animals preferentially explored the novel rather than the familiar one (Bonferroni’s *post hoc* test: *P* < 0.001). Accordingly, the discrimination index was significantly lower than controls (*t*-test: *P* < 0.05; Figure 3B). No differences in total exploration time were found between experimental groups (*t*-test: n.s.; Figure 3C).

### Effects of memantine on long term memory impairment induced by acute i.c.v. injection of sAβ

**Figure 4:**
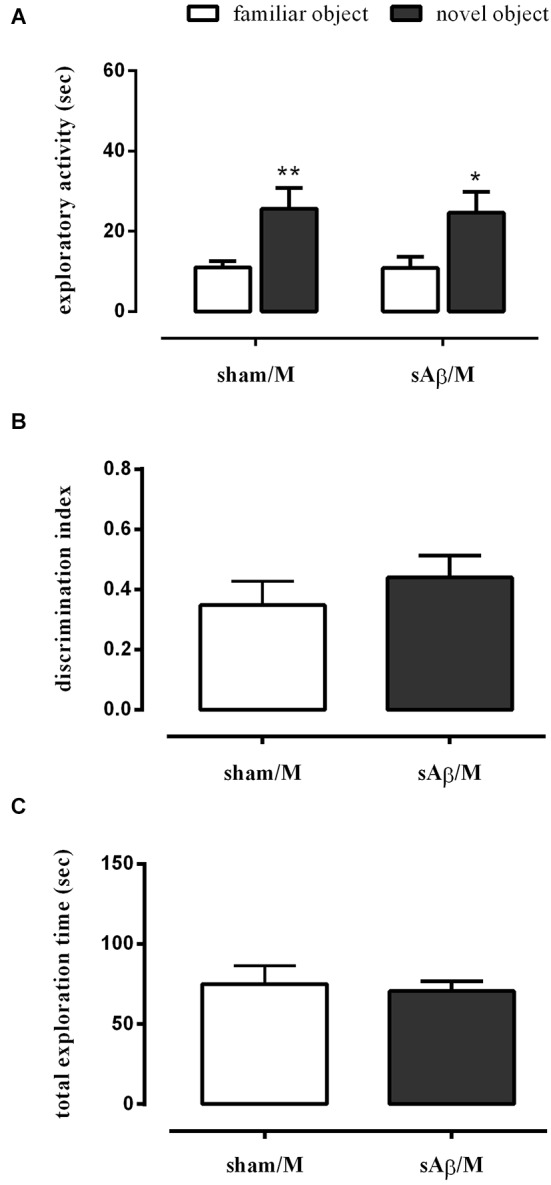
**(A)** Exploratory activity of novel and familiar objects during T2, **(B)** discrimination index and **(C)** total exploration time in NOR2 test. Animals received i.c.v. injection of vehicle (5 μl; sham) or sAβ (4 μM, 5 μl) 2 h before training (T1) and tested 24 h later (T2). Memantine (5 mg/kg) was given immediately after a 10 min training. Data are expressed as mean ± SEM of total time in s (*n* = 8 sham/M; *n* = 8 sAβ/M). (Two-way RM ANOVA followed by Bonferroni’s multiple comparisons test **P < 0.01 and *P < 0.05 vs. familiar object).

To establish whether the memory deficit observed in the NOR2 protocol was dependent on glutamatergic modulation, rats were treated with memantine (5 mg/kg i.p.) or vehicle immediately after the 10 min training session (T1). Twenty-four hours later, rats were subjected to the test phase, as described above. As shown in Figure 4A, a clinically relevant dose of memantine was able to prevent the long term memory impairment induced by sAβ (Two-way RM ANOVA followed by Bonferroni’s multiple comparisons test: *Ft*_(1,14)_ = 23.34, *P* < 0.001). Then, while rats injected with sAβ did not discriminate between the familiar and the new object, the inhibition of NMDA receptors prevented this memory deficit (*t*-test: n.s.; Figure 4B). No differences in total exploration time were found between experimental groups (Figure 4C).

**Figure 5:**
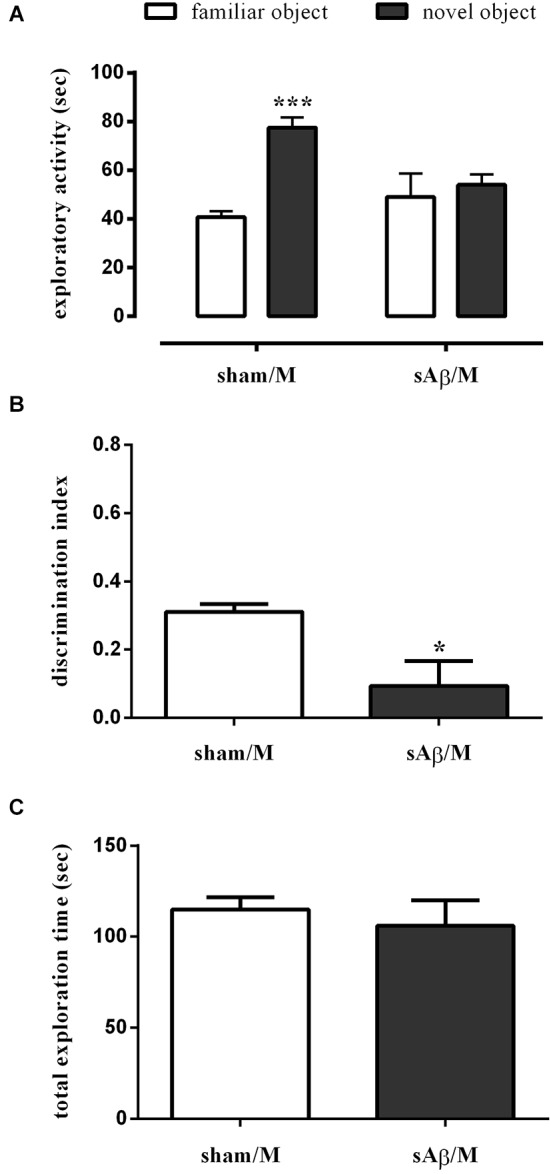
**(A)** Exploratory activity of novel and familiar objects during T2, **(B)** discrimination index and **(C)** total exploration time in NOR2 test. Animals received i.c.v. injection of vehicle (5 μl; sham) or sAβ (4 μM, 5 μl) 2 h before training (T1) and tested 24 h later (T2). Memantine (5 mg/kg) was given 30 min before training. Data are expressed as mean ± SEM of total time in s (*n* = 6 sham/M; *n* = 6 sAβ/M). (Two-way RM ANOVA followed by Bonferroni’s multiple comparisons test ***P < 0.01 vs. familiar object, *t*-test *P < 0.05 vs. sham).

Interestingly, when memantine was administered 30 min before the familiarization phase (T1), sAβ-injected rats, tested 24 h later, remained cognitively impaired (Two-way RM ANOVA followed by Bonferroni’s multiple comparisons test: *Ft*_(1,10)_ = 25.13, *P* < 0.001; Figure 5A). Accordingly, the discrimination index was significantly lower than control (*t*-test: *P* < 0.05; Figure 5B). Total exploration time was not modified between experimental groups (*t*-test: n.s.; Figure 5C). The treatment with memantine alone did not affect memory.

### Effects of acute i.c.v. injection of sAβ on glutamate levels

Glutamate basal levels were measured in microdialysis fluid 2 h after i.c.v. administration of sAβ or vehicle in PFC, no more than 10% difference among sample was found, then the data obtained from four consecutive samples per animal were averaged, and mean value from *n* animals per group was determined.

**Figure 6:**
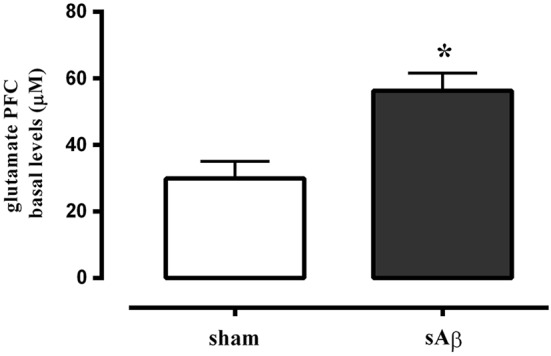
**Quantitative analysis of basal extracellular glutamate levels in PFC of rats 2 h after i.c.v. injection of vehicle (5 μL; sham) or sAβ (4 μM, 5 μl).** Data are expressed as mean ± SEM (*n* = 6 sham, *n* = 4 sAβ). (Student *t*-test *P < 0.05).

As shown in Figure 6, extracellular levels of glutamate were significantly increased in PFC of sAβ-treated animals compared to control (*t*-test: *P* < 0.05).

## Discussion

Our results show for the first time that an acute i.c.v. injection of sAβ peptide, during the process of long term memory formation, did not disrupt memory storage but, when the information was properly stored, it acutely interfered with its consolidation/retrieval, and such effect was prevented by NMDA receptor inhibition. The possibility to mimic the detrimental action of sAβ peptides *in vivo* provides a very interesting tool for understanding their mechanism of action. However, since a single species of Aβ is responsible for the complex dysregulation of intracellular signaling pathways that takes place in Aβ-mediated effects, it is reasonable to expect that distinct Aβ assemblies may differentially affect specific pathways underlying synaptic dysfunction (Mucke and Selkoe, 2012). Moreover, given that Aβ oligomeric species act on independent targets, it seems of particular interest to understand the biological effects of a particular assembly form. Unlike Aβ assemblies purified from brain’s patients, commercial available Aβ fragments are considered useful tools as they are chemically defined, and can be easily characterized, from a structural point of view, by using several techniques, ranging from western blot (Jin et al., 2011) to nuclear magnetic resonance (Yu et al., 2009) and electrophoresis (Wiltfang et al., 1997). However, one of the best and most informative technique is atomic force microscopy (AFM; Kowalewski and Holtzman, 1999; Balducci et al., 2010), which provides a high-resolution image at the molecular level revealing details of protein structures. By using AFM, we precisely characterized sAβ fragments and, to further confirm the conformation of the peptide, we used the transmission electronic microscopy (TEM); both techniques enabled us to feature the types of sAβ fragments, with no fibrils being present in the injected solution (our unpublished data, Campagna et al., 2011). Moreover, in our study, the use of defined synthetic sAβ preparations removed unknown factors, which can be present in cells and brain extracts or cerebrospinal fluid that could mask or exacerbate their effects. From a behavioral point of view and to explore the effects of sAβ on memory, we chose the NOR test. This behavioral paradigm has become a widely used model for the investigation in memory alterations. It is usually used to measure recognition memory, which is heavily impaired in AD. This test represents a simple and reproducible behavioral assay, which relies primarily on rat’s innate and spontaneous exploratory behavior in the absence of reinforcement or the need for stressful elements. It has been demonstrated that the NOR test allows to study either short or long term memories. In particular, results of NOR paradigm are influenced by the manipulation of the amount of time required for the intertrial interval (Taglialatela et al., 2009). In the present study, two variants of NOR test were used to investigate the effects of sAβ on short and long term memory. During the NOR1, animals were exposed to the test phase 1 min after the familiarization phase. During testing, a novel object needs to be detected and encoded, while a familiar object needs to be updated. In these experimental conditions, rats treated with sAβ 2 h before behavioral investigation, exhibited normal performance during the test and remained cognitively intact. In these animals, when novel and familiar stimuli were present together, the novel stimulus was more explored until novelty was lost. Indeed, the preference for the novel object means that presentation of the familiar one was already present in their memory, indicating that during the NOR1, sAβ did not affect this short term memory. Then, in our animal model, sAβ injected i.c.v. before the acquisition of the information (familiarization phase) did not affect the information being encoded. These results confirmed our previous investigations where we demonstrated that, in the context of normal levels of general motor activity, sAβ did not alter the ability of rats to differentiate between a familiar and a novel object, in a short term memory test, even when the test was performed 7 days after i.c.v. injection (Colaianna et al., 2010). However, memory relies on a set of processes by which information is encoded, consolidated, and retrieved. In addition, when a certain memory is recovered in the presence of novelty, it is set into an unstable state, during which its retention can be either enhanced or impaired.

Over the next hours, memories require stabilization to persist and are consolidated over time into long term memories, and once in that state, they remain fixed. This processing memory is involved in the reorganization of the already formed memories, allowing then incorporation of new information (Clarke et al., 2010). Based on this, although the NOR1 behavioral protocol could not permit to assess sAβ effects on consolidation or recall processes, it seems that the peptide does not affect short term memory. Thus, we modified the retention interval, which is the amount of time the rat must retain the memory of the two identical objects presented during the training session prior to the testing session. Thus, the rat’s ability to acquire the object recognition memory by using an intertrial interval of 24 h was tested. Interestingly, in these experimental conditions (NOR2), when sAβ-treated rats were re-exposed to either the familiar or the novel object, they did show significant difference between groups in time spent for exploration of both objects, meaning that animals were unable to remember the object previously investigated. These data suggest that, when the information was properly encoded, as demonstrated by using the NOR1 task, sAβ did not disrupt memory storage but acutely interfered with its consolidation/retrieval. Actually, disruption of memory formation results from interference with key biological mechanisms underlying the stabilization of memory.

Depending on the time when the disturbance occurs in this biological activity, a specific phase of memory formation might be influenced. The delay-dependent decrease in memory recognition we observed might result from a decay in memory of the familiar object. Thus, one hypothesis that could account, at least in part, for these results is that sAβ was able to induce anterograde amnesia, namely the inability to learn new information, although this hypothesis requires further investigation. It is known that patients with this form of amnesia preserve intact the capacity to retain small amounts of information over short periods of time but are dramatically impaired in their ability to form long-term memories (Markowitsch and Staniloiu, 2012). Thus, in our animal model, a single i.c.v. injection of sAβ impaired memory consolidation/retrieval within 24 h, suggesting that the peptide interferes with the synaptic activity, which is essential for the stabilization of new memories (Mayford et al., 2012). Indeed, if the memory was properly consolidated in the rats, then it should have been retrievable during the test phase, as seen in controls. Thus far, there are only few *in vivo* studies of the involvement of sAβ in memory alterations in rats (Cleary et al., 2005; Lesné et al., 2006; Poling et al., 2008; Shankar et al., 2008). As demonstrated by Lopez-Ramos et al. (2012), SAMP8 mice showing cognitive dysfunctions probably related to amyloid aggregates have significant deficits in the acquisition phase of the object recognition test. Our results seem in some contrast to the evidence reported in AD patients, who are unable to store newly acquired information but preserve old memories, especially in early-stage of the disease. However, based on our results, it cannot be excluded that, in AD patients, the old events have occurred before the brain damage and this made possible the retention of retrieved material which has been successfully consolidated and stored into old memories for such events. On the other hand, in regard to the ability of sAβ to impair memory, possibly by inhibiting LTP, it has been hypothesized that the peptide may play a role in the process of physiological forgetfulness (Hasegawa, 2007). Interestingly, pathologies in which this physiological process seems to be impaired, such as posttraumatic stress disorder, are linked to increased risk of cognitive decline and AD-associated dementia (Weiner et al., 2014). The mechanism by which sAβ peptide induces consolidation/retrieval impairment is not clear yet, but the present results raise the possibility that the modulation of glutamatergic neurotransmission could be involved. Indeed, memory processing requires glutamatergic receptor activation (Kandel et al., 2014). It has been recently demonstrated that sAβ dimers extracted from AD cortex induced their effects by perturbing glutamatergic synaptic transmission, being mGlu receptors required for the induction of LTD, while NMDA receptors were needed for spine loss (Shankar et al., 2008). Moreover, an association of Aβ peptides with several receptors has been described in literature, and these receptors might act as substrates for the Aβ-mediated detrimental effects leading to the aforementioned functional cognitive alterations. Among others, glutamatergic receptors have been proposed as mediating the effect of Aβ on synaptic plasticity and memory (Klyubin et al., 2008; Ferreira and Klein, 2011). Consistent with this, sAβ enhances NMDA receptor agonist-induced delayed cognitive dysfunction (Dornan et al., 1993; Nakamura et al., 2006). Soluble Aβ has also been found to enhance glutamate release from microglia (Noda et al., 1999), isolated brain nerve terminals (Bobich et al., 2004) and brain slices (Arias et al., 1995; Chin et al., 2007; Puzzo et al., 2008; Kabogo et al., 2010). Accordingly, in our experimental conditions, we found a significant increase in glutamate release in PFC of treated rats. Such result is of particular interest in our research considering that it has been reported that PFC plays a crucial role in object recognition as well as in memory consolidation (Ragozzino et al., 2002; Xiang and Brown, 2004). Moreover, blocking synthesis of protein or NMDA receptors resulted in failure of consolidation of memory 24 h after familiarization phase (Akirav and Maroun, 2006). On the other hand, several reports suggest that NMDA receptors were closely associated with Aβ oligomer binding sites on neurons (De Felice et al., 2007; Lacor et al., 2007; Dewachter et al., 2009). Then, given the role of NMDA receptors in Aβ-induced synaptic dysfunction, we directly tested the hypothesis that a NMDA receptor-blocking dose of memantine (5 mg/kg i.p.), clinically used to treat AD, could counteract the consolidation/retrieval failure observed in sAβ-treated rats. Memantine is an NMDA receptor open channel blocker, which has been found to act with a low-to-moderate affinity at therapeutic concentrations (Lipton, 2007; Parsons and Gilling, 2007; Parsons et al., 2007). Unlike many high affinity antagonists (Ikonomidou and Turski, 2002), it preferentially blocks excessive or inappropriate activation of NMDA receptors, while leaving physiological NMDA receptor-mediated activity unaffected (Lipton, 2007; Parsons and Gilling, 2007; Parsons et al., 2007). *In vitro* and *in vivo* studies using animal models of neurodegenerative diseases and stroke showed that memantine protects neurons from NMDA receptor-mediated excitotoxic damage (De Felice et al., 2007; Xia et al., 2010). Mechanistically, we found that acute treatment with NMDA receptor blocking dose of memantine can prevent the consolidation/retrieval failure, caused by exogenous acute sAβ administration. Accordingly, these results confirm previous data showing that sAβ oligomers directly activate NMDA receptors (Texidó et al., 2011). It should be noted that the observed effects of memantine in the present study were achieved at concentrations that are considered clinically relevant, leading to plasma levels of 1.2 μM, and that memantine has been shown to be active as an NMDA receptor antagonist at this concentrations as well (Misztal et al., 1996; Danysz and Parsons, 2003). Of note, when memantine was given before encoding memory process, animals remained cognitively impaired, suggesting that, in presence of exogenous sAβ, excessive NMDA activation, due to increased glutamate release, is deleterious only during the consolidation/retrieval phase. On the other hand, it has been demonstrated that, if acute administration is used, than memantine 5 mg/kg i.p., is the maximal therapeutically relevant dose for 30–60 min time point, although age, strain, gender and health status of rats should also been considered, since they can influence pharmacokinetic parameters considerably (Danysz et al., 1997). In addition, it has been reported that such dose took the peak serum concentration at *ca*. 30 min, which correspond to the upper limit of dose seen in patients and volunteers after therapeutic dose of memantine (Zoladz et al., 2006). It is important to underline that, under our experimental conditions, memantine, given alone or co-administered with sAβ had no effect on locomotor (data not shown) and exploratory activities. Our findings are in good agreement with clinical results showing that memantine treatment improves cognitive performance in AD patients (Peskind et al., 2006). In addition, memantine administration has been previously associated to an improvement in cognitive performance in animal models of AD, characterized by amyloid deposition (Nakamura et al., 2006; Martinez-Coria et al., 2010). The novelty of our findings relies on the evidence that memantine was effective in the absence of actual Aβ deposits. In this regard, we have previously demonstrated, by immunofluorescence experiments, that after 48 h from i.c.v. injection, sAβ peptide was no longer detectable in the ventricular space (Trabace et al., 2007). Then, it seems likely from our results that sAβ-induced impairment of consolidation/retrieval of long term memories and memantine ability to restore cognitive deficits might precede the development of insoluble amyloid plaque cores and then neurodegeneration. Our data also indicate that, in this model, acute sAβ-mediated memory impairment is not dependent on a persistent neurodegenerative phenomenon and can be rescued, suggesting that early targeting sAβ-induced alterations might lead to the recovery of cognitive impairment.

## Conclusions

Our study showed that sAβ was able to acutely alter consolidation/retrieval of long term memory, and this effect suggesting that early targeting sAβ-induced alterations might lead to the recovery of cognitive was prevented by the use of a clinically relevant dose of memantine. This evidence suggests that sAβ-induced consolidation/retrieval failure might contribute to the memory impairment observed in AD even in the early stages of disease. Moreover, the present findings further our knowledge on the possibility of prevent cognitive deficits with NMDA receptor inhibition in an acute rat model of i.c.v. sAβ injection. Besides, using the acute animal model to explore the mechanism of action of sAβ, it might also be helpful to assess the efficacy of novel therapeutic approaches directly aimed at this soluble species.

## Conflict of interest statement

The authors declare that the research was conducted in the absence of any commercial or financial relationships that could be construed as a potential conflict of interest.
